# Patient-reported burden in adults with atopic dermatitis: an international qualitative study

**DOI:** 10.1007/s00403-024-03130-w

**Published:** 2024-06-08

**Authors:** Andreas Wollenberg, Melinda Gooderham, Norito Katoh, Valeria Aoki, Andrew E. Pink, Yousef Binamer, Jonathan I. Silverberg

**Affiliations:** 1 Department of Dermatology and Allergy, Ludwig-Maximilian University of Munich, Munich, Germany; 2 Department of Dermatology, Augsburg University Hospital, Augsburg, Germany; 3 Department of Dermatology, Queen’s University, Ontario, Canada; 4 Department of Dermatology, SKiN Centre for Dermatology, Ontario, Canada; 5 Department of Dermatology, Kyoto Prefectural University of Medicine, Kyoto, Japan; 6 Department of Dermatology, University of São Paulo School of Medicine, São Paulo, Brazil; 7 St. John’s Institute of Dermatology, Guy’s & St. Thomas’ NHS Foundation Trust, London, UK; 8 Department of Dermatology, Alfaisal University, Riyadh, Saudi Arabia; 9 Department of Dermatology, King Faisal Specialist Hospital & Research Centre, Riyadh, Saudi Arabia; 10 School of Medicine and Health Sciences, George Washington University, Washington, DC USA

**Keywords:** Atopic dermatitis, Patient research, Patient-reported outcomes, Quality of life, Qualitative research

## Abstract

The objective was to study a large, international, ethnically diverse population of patients with atopic dermatitis (AD) to support the creation of patient-centric recommendations for AD management. Qualitative data were generated from 45-min, 1:1 telephone interviews conducted across 15 countries in each patient’s native language. Interviews explored the impact of AD on patients’ lives, patients’ most important symptoms, treatment expectations, and treatment decision-making. Participants were also questioned on their current knowledge of AD scoring systems and what was most important to include in these tools. In total, 88 adult patients (≥ 18 years old) receiving treatment for AD were recruited through a market research database, clinician referrals, and local advertising. All patients were screened to ensure a balanced and diverse sample in terms of age, gender, educational level, employment status, geographic location, and AD severity. Patients involved in market research or activities supporting advocacy groups within the previous 6 months or affiliated with or employed by pharmaceutical companies were excluded. AD had a substantial impact on patients’ lives. Itch, skin redness, and dry/flaky skin were the most frequently reported symptoms, with > 75% of patients experiencing these symptoms every 1–3 days. Mental health issues were common and resulted in the greatest negative impact on patients’ daily lives. Patients perceived clinicians to underestimate the burden of their AD. Patients had little awareness of AD scoring systems and indicated a preference for these to be more clearly incorporated in clinical practice. For an ideal scoring system, patients favored using a combination of patient-reported and clinician-reported outcomes to reflect disease burden and ensure consistency across all settings. This global study generated diverse patient perspectives on the disease burden of AD, their expectations of treatment, and their views on AD scoring methods. These data provide evidence to support the development of patient-centric recommendations for AD management.

**Supplementary Information:**

The online version contains supplementary material available at 10.1007/s00403-024-03130-w.

## Introduction

Atopic dermatitis (AD) is a chronic skin disease characterized by inflamed, dry, itchy skin [1]. Until recently, AD was viewed as a disease that only affected the skin [2]. However, psychological symptoms such as sleep disturbance, depression, and anxiety are among the most common manifestations among patients with AD [3]. Moreover, National Health and Wellness Survey (NHWS) data from Europe show that patients with AD, particularly those with inadequately controlled disease, have a high disease burden, with many impacts on physical function and reduced quality of life [4]. In addition, patients with moderate-to-severe AD suffer a higher prevalence of atopic and psychological comorbidities, lower work productivity, and increased activity impairment compared with patients without AD [4]. NHWS data from the USA [5] and Japan [6] showed similar findings, while data from the European NHWS revealed that patients suffering from mild-to-moderate AD also have a high disease burden; their most common comorbidities were consistent with those experienced by patients with more severe disease [7]. These findings highlight the humanistic burden of AD and the unmet need for improved care standards across all disease severities.

However, qualitative research into how patients make treatment decisions, their treatment expectations, and their views on treatment targets and scoring systems is currently limited. While some studies highlight the burden of AD, they predominantly focus on a specific country or region, and do not clearly capture the multidimensional impact [4, 5, 7, 8]. Moreover, most AD management guidelines lack the evidence base to adequately incorporate global patients’ views into recommendations [9, 10], although more recent guidelines such as the EuroGuiDerm guidelines for AD management contain a dedicated patient’s view section. [11].

In 2021, a treat-to-target initiative attempted to address gaps in AD management by developing a clinical decision-making model to support clinicians in their choice of treatment targets. While patients were involved in voting on the strength of each recommendation, they were not involved in developing the recommendations [12]. Qualitative patient research is, therefore, needed to create truly patient-centric AD assessment and management recommendations.

## Patients and methods

### Patients

Adult patients (≥ 18 years old) receiving treatment for AD were recruited through a market research database, clinician referrals, and local advertising. Potential candidates were screened for eligibility; questions focused on the patient’s gender, age, educational level, time since AD diagnosis, types of consultation with clinicians in the past 12 months, current treatment types, and employment status, providing a diverse range of background information. Patients were required to have a diagnosis of and be currently receiving treatment for AD. Information on AD severity was obtained during screening using the Patient-Oriented Eczema Measure (POEM) scoring tool, although patients were unaware that they were completing a POEM questionnaire. Those involved in market research or activities supporting advocacy groups within the previous 6 months were excluded. Any patients affiliated with or employed by pharmaceutical companies as a consultant and/or researcher were also excluded.

### Telephone interviews

Each patient participated in a 45-min, 1:1 telephone interview conducted in their native language (Arabic, Chinese, English, French, German, Italian, Japanese, Polish, Portuguese, Russian, or Spanish) by a specialist healthcare market research team. These interviews were structured and focused on key areas relating to AD management. Most questions were free-form, with a few multiple-choice questions. Screen sharing was used during interviews to aid patients’ understanding of the questions and assess their responses. All interviews were audio recorded, and secondary (content) analysis was conducted in a Word document for each market.

After a brief introduction, patients were asked questions on the day-to-day impact of the disease on their quality of life. Participants were asked to rank their most troublesome symptoms; options included itch, skin redness, dry skin, pain, sleep disturbance, mental health issues (e.g. anxiety or depression), treatment burden, and skin dyspigmentation. To gain patient feedback on disease/symptom severity scoring systems, a list of 13 scoring systems was agreed upon by the authors. The scoring systems were chosen to include those that are most frequently used in clinical practice and to cover a wide range of atopic and psychological AD symptoms with both patient- and clinician-reported measures included (SCORing Atopic Dermatitis [SCORAD], Eczema Area and Severity Index [EASI], body surface area, Investigator’s Global Assessment, Dermatology Life Quality Index, itch Numeric Rating Scale [NRS], sleep NRS, pain NRS, POEM, Work Productivity and Activity Impairment, Multimorbidity Treatment Burden Questionnaire, Rajka–Langeland score, and patient-reported global AD severity). For feasibility, each respondent was shown five different scoring systems via screen sharing; interviewers provided a brief, patient-friendly explanation of each of the systems, and patient perspectives on each scoring method were explored (Table S1; see Supporting Information). Patients were asked to rank each scoring system according to its relevance to their needs.

Patients were also questioned on how they make treatment decisions and what triggered their most recent change in therapy. In addition, the interviewers collected feedback on the relationship dynamic that patients had with their clinicians and patients’ perceived level of involvement in the treatment decision-making process. Finally, patients discussed their treatment expectations, how these expectations may have differed from reality, and how satisfied they were with their current treatment regimen.

### Patient and public involvement

Participants were not involved in the design of the study or recruitment, but participated in qualitative interviews as described above.

## Results

### Patient characteristics

In total, 88 patients from 15 countries were included in the study. The largest proportion of patients were from the USA (19.3% [*N* = 17]); six (6.8%) were from the UK, and five (5.7%) were from each of Belgium, Brazil, Canada, China, France, Germany, Italy, Japan, Mexico, Poland, Russia, Saudi Arabia, and Spain (Table 1). The largest patient age group (37.5%) was 30–44 years (*N* = 33) and over half (62.5%) of all patients were female (*N* = 55). The majority of patients had completed secondary school (91% [*N* = 80]). Most patients had previously seen a dermatologist for their AD (92% [*N* = 81]), and over one-third of patients had seen a primary care provider (35.2% [*N* = 31]). Patients also varied in their POEM-assessed AD severity, which was mild for 11, moderate for 33, severe for 33, and very severe for 11.

**Table 1:** Characteristics of the global patient population (*N* = 88)

Patient characteristics	*N* (%)
Gender identity	
Male	33 (37.5)
Female	55 (62.5)
Age (years)	
18–29	22 (25.0)
30–44	33 (37.5)
45–59	19 (21.6)
≥ 60	14 (15.9)
Country of residence	
USA	17 (19.3)
UK	6 (6.8)
Canada	5 (5.7)
Germany	5 (5.7)
France	5 (5.7)
Italy	5 (5.7)
Spain	5 (5.7)
Belgium	5 (5.7)
Japan	5 (5.7)
China	5 (5.7)
Saudi Arabia	5 (5.7)
Russia	5 (5.7)
Poland	5 (5.7)
Mexico	5 (5.7)
Brazil	5 (5.7)
Highest level of education attained	
Did not finish grade/primary/elementary school	2 (2.3)
Grade/primary/elementary school	6 (6.8)
High school/secondary school	40 (45.5)
College/university-level education or higher	40 (45.5)
Healthcare practitioner(s) seen^a^	
Dermatologist	81 (92.0)
Nutritionist	2 (2.3)
Allergist	10 (11.4)
Dermatology nurse	4 (4.5)
PCP	31 (35.2)
Naturopath/TCM	4 (4.5)
AD severity (POEM)^b^	
Mild	11 (12.5)
Moderate	33 (37.5)
Severe	33 (37.5)
Very severe	11 (12.5)

*AD* atopic dermatitis, *PCP* primary care provider, *POEM* Patient-Oriented Eczema Measure, *TCM* traditional Chinese medicine

^a^Patients were able to select more than one healthcare practitioner

^b^Patients were unaware that they were completing a POEM questionnaire

### Impact of AD on patients’ lives and the most troublesome symptoms

**Fig. 1:**
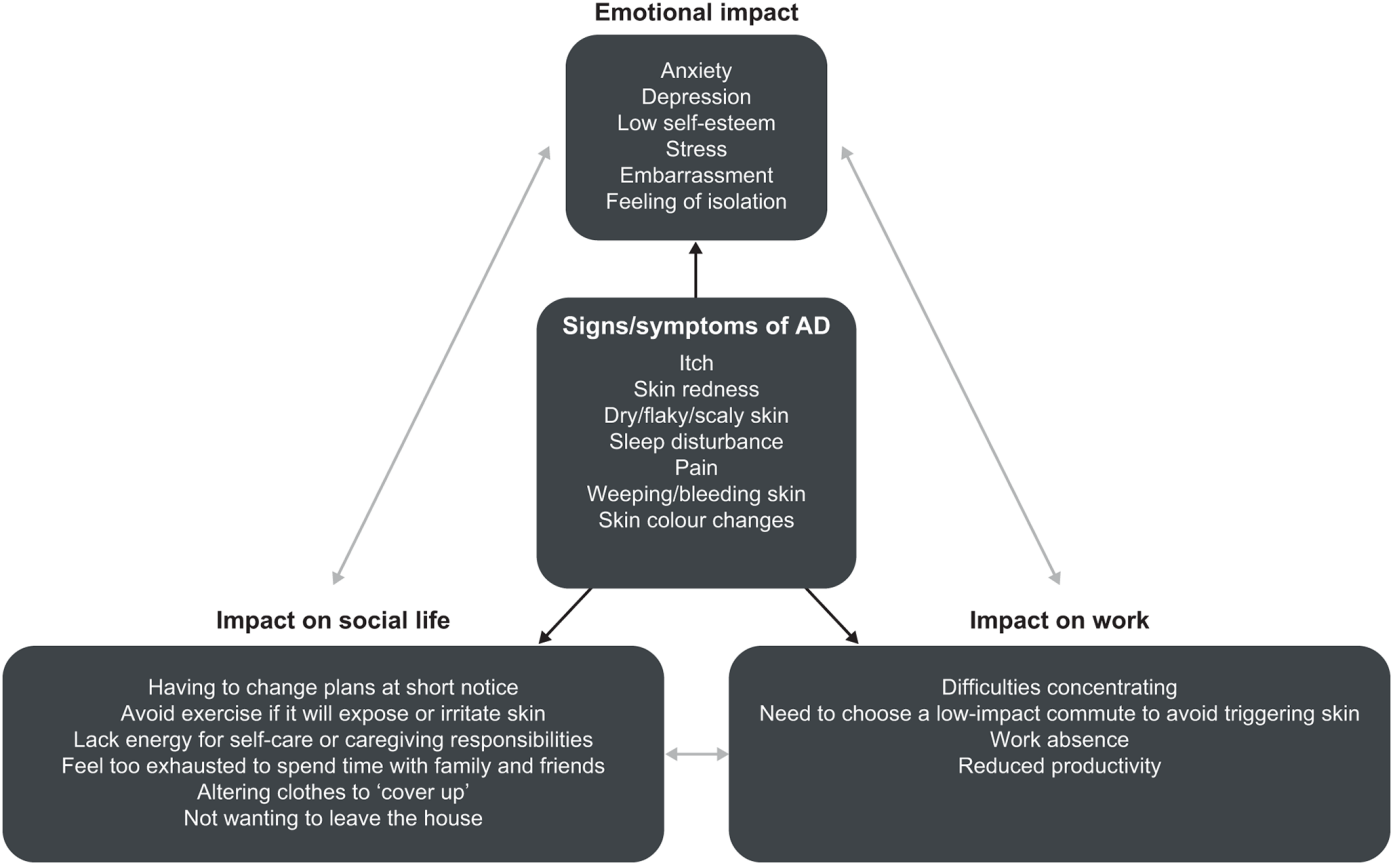
Summary of the impact of AD on patients’ lives^a^. *AD* atopic dermatitis. ^a^Black arrows indicate a direct impact of signs/symptoms of AD on patients; grey arrows indicate indirect impacts on emotional, social, and work aspects of patients’ lives

AD had a substantial, broad impact on patients’ lives, with emotional, social, and work effects, as summarized in Fig. 1 (see also Table S2). Patients revealed that they felt impacted by their AD at all times of the day and night. In the mornings, patients reported feeling tired due to poor sleep and revealed how their AD required them to use special body washes and spend extra time showering and moisturizing. Furthermore, patients reported that their AD affected their choice of clothing, and that they may need to take alternative, low-impact commutes to work to avoid triggering their skin symptoms. When they arrived at work, patients reported difficulties with concentration, and after work, patients sometimes felt too exhausted to spend time with family and friends and lacked the energy for self-care or caregiving responsibilities. In the evening, patients said they may experience anxiety about a potential lack of sleep, and reported spending extra time moisturizing and preparing for bed.

Patients described their AD as a lonely experience and were reluctant to share details of their disease due to feelings of embarrassment and concerns about being “judged.” Many patients underestimated the impact of AD on their daily lives, as they realized that the emotional and physical impact of AD was more widespread than they initially thought when prompted to give additional details during interviews. Patients revealed that AD affects their life choices, results in them needing to adjust or cancel plans, influences their wardrobe choices, and requires them to take time off work. AD was viewed as an “aggressive” condition that often lies in wait. Many of the patients had experienced exacerbations, which they defined as periods of time where they were unable to control their symptoms with their usual AD routine, and consequently experienced negative impacts on their quality of life. Most patients were either concerned about future exacerbations or resigned to the understanding that they would happen. Fear of exacerbating AD symptoms caused many patients to avoid certain foods, with some removing sugar, dairy, gluten, and/or alcohol from their diet.

**Fig. 2:**
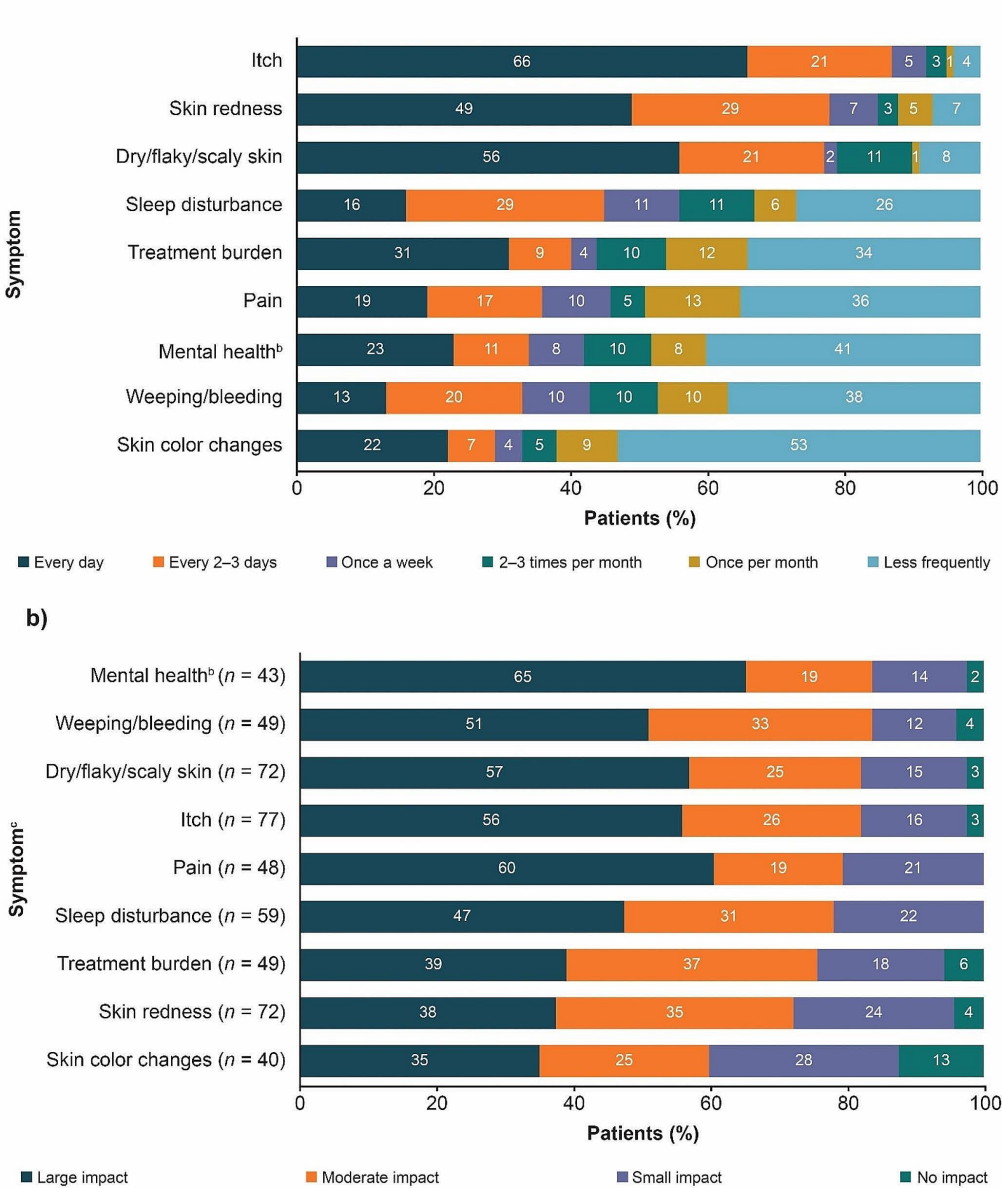
Frequency of AD symptoms reported by patients (**a**) and impact of AD symptoms on patients’ daily lives (**b**) (*N* = 88)^a^. *AD* atopic dermatitis. ^a^Percentages may not add up to 100 due to rounding. Order based on combined total of first and second responses to respective questions. ^b^For example, anxiety or depression. ^c^From respondents who experienced the symptoms at least once per month

Itch, skin redness, and dry/flaky/scaly skin were the most frequently reported symptoms, with over 75% of patients suffering every 1–3 days (Fig. 2a). Itch was the primary symptom experienced across patients from all regions, with two-thirds of patients (66%) reporting daily itch. Sleep disturbance was common, with 45% of patients suffering from a lack of sleep every 1–3 days.

For many patients, the cumulative burden of AD led to mental health issues such as lack of confidence, anxiety, and depression. More than half of patients (52%) said they suffered from mental health issues such as anxiety or depression at least 2–3 times a month, with 23% stating that they suffer from mental health issues daily. Almost one-third (31%) suffered daily symptoms relating to treatment burden, with two-thirds (66%) suffering at least once monthly. Mental health issues had the worst impact on patients’ lives (Fig. 2b). Of patients who experienced AD symptoms at least once a month, 84% stated that mental health issues had at least a moderate impact on their lives, with 65% considering them to have a large impact. Only 2% of patients stated that mental health issues relating to their AD had no impact on their lives. Physical symptoms such as weeping/bleeding skin, dry/flaky/scaly skin, itch, and skin pain were deemed to have at least a moderate impact in over 79% of patients, with over half of patients (≥ 51%) ranking these symptoms as having a large impact on their lives.

### How patients make treatment decisions

**Fig. 3:**
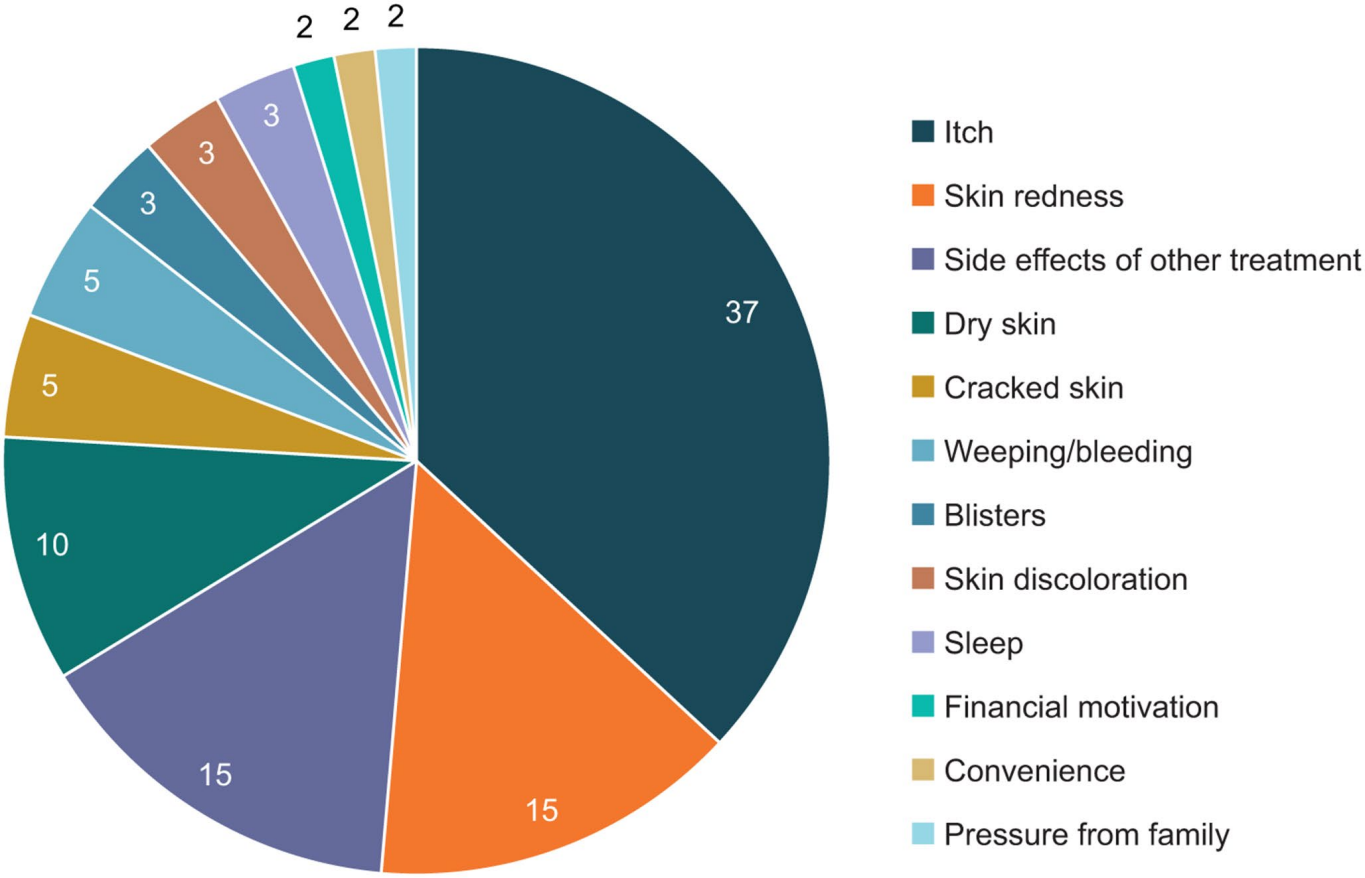
Patients’ drivers for requesting a change in treatment from their clinician (*n* = 66) ^a^. Percentages may not add up to 100 due to rounding. ^a^ Respondents who provided a reason for requesting a change in treatment

Patient interviews revealed that 75% of patients had previously requested a change in medication from their clinician. Itch was found to be the main driver for patients requesting a change in treatment, being cited by 37% (Fig. 3; see also Table S2).

Skin redness and side effects associated with other treatments were the next most common reasons for requesting a change (15% each). Pressure from family, financial motivation, and treatment convenience were the lowest reported drivers for wanting to change medication (only 2% each).

### Patients’ treatment expectations

Patients stated that they perceive AD treatments to be “short-term fixes” and felt frustrated with their lack of long-term efficacy (see also Table S2). In addition, patients revealed that their expectations for a treatment were correlated with the treatment burden: treatments with higher frequencies of administration, more intense side effects, or more invasive procedures (i.e. injections) were associated with higher expectations from patients.

Patients reported that they expected treatments to alleviate symptoms to the point that they are able to sleep through the night and no longer have to change their clothing or alter their plans due to AD, while acknowledging that they will still experience some degree of itch, dry skin, and/or skin redness.

Patients did not have a clear or consistent definition of symptom improvement. While patients did not expect their condition to be completely cured, the levels of improvement viewed as significant varied, depending on factors such as the severity of their AD and symptoms.

### Patients’ communication with their clinicians

While many patients stated that they had good relationships with their clinicians, some felt that clinicians underestimated their disease burden; this was reported more often for non-specialists than dermatologists. There was some concern that AD was viewed as only a skin condition, not appreciating its broader impact on patients’ lives. Some patients expressed concern that their clinicians see them as “just another” patient with AD and suggested that clinicians may have become desensitized to their disease burden. Patients also reported that they are often unable to see clinicians when their symptoms are at their most burdensome. Furthermore, a perceived lack of “caring” or knowledge from non-specialist clinicians was cited by some patients as a reason to look for alternative treatments (e.g. traditional Chinese medicine, alternative diets).

Patients also felt they were not given enough time to express themselves in medical appointments and reported an inability to communicate optimally with their clinicians (Table S2). Many patients felt that they were not listened to by their clinicians, stating that consultations can sometimes feel “like a waste of time,” resulting in them feeling isolated, frustrated, and demotivated to manage their AD.

Patients trusted clinician decisions across all regions; however, perceived patient involvement in treatment decisions varied. For example, in the USA, Latin America, Western Europe, Canada, Japan, and Asia, primary treatment decisions were perceived to lie in the hands of the clinician. By contrast, in Eastern Europe, the Middle East, and Africa, treatment decisions were viewed as more collaborative. Moreover, differences were observed within regions. For example, in Asia, while patients in China were more passive and happier with clinicians making primary treatment decisions, patients in Japan wanted to take a more active role in their AD management. In Western Europe and Canada, patients in the UK, Spain, Germany, France, and Italy felt more involved in treatment decisions than patients from Belgium and Canada. However, all patients from these countries still perceived clinicians to be “in charge.” Patients revealed that a good patient–clinician relationship involves a clinician who collaborates, shares their knowledge, and comforts the patient, with the patient feeling understood.

### Patients’ views on AD scoring systems

Overall, patients were unfamiliar with existing scoring systems, with only a minority recognizing any of the named systems. Few patients reported using scoring systems during previous healthcare appointments; a minority of patients mentioned using “questionnaires” relating to primarily physical symptoms during consultations (see also Table S2).

Scoring systems were perceived to be clinician-centric, helping clinicians to evaluate patients’ skin rather than assisting patients in communicating their needs. When questioned about their preferences for different AD scoring systems, patients favored using a combination of patient-reported outcomes to reflect disease burden and clinician-reported outcomes to prevent patients overestimating the severity of their symptoms.

**Fig. 4:**
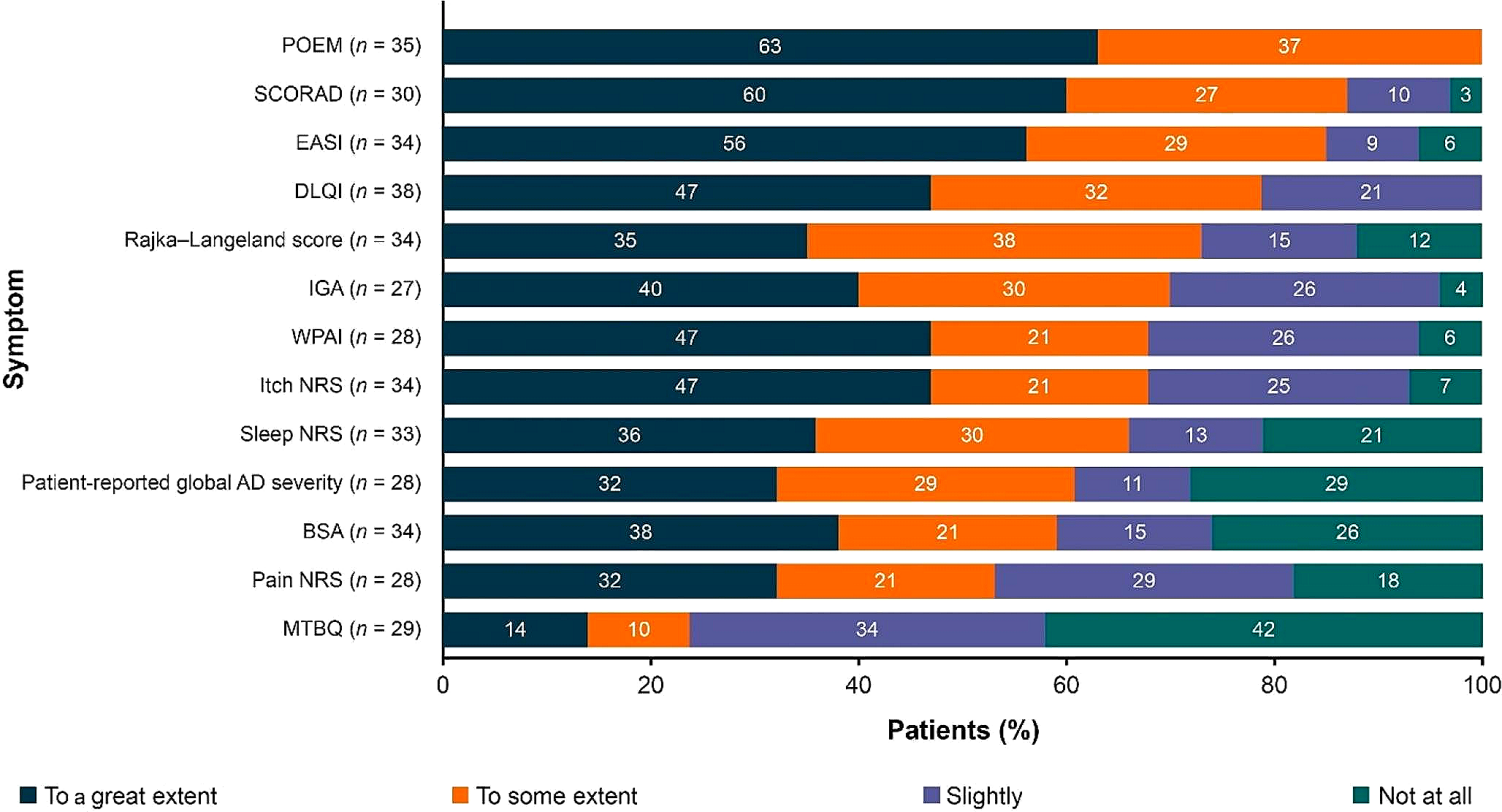
Patients’ perceptions of current AD scoring systems (*N* = 88)^a,b^. *AD* atopic dermatitis, *BSA* body surface area, *DLQI* Dermatology Life Quality Index, *EASI* Eczema Area and Severity Index, *IGA* Investigator’s Global Assessment, *MTBQ* Multimorbidity Treatment Burden Questionnaire, *NRS* Numeric Rating Scale, *POEM* Patient-Oriented Eczema Measure, *SCORAD* SCORing Atopic Dermatitis, *WPAI* Work Productivity and Activity Impairment. ^a^Each respondent was shown five different scoring systems and was asked, “Based on what’s included in the scale, how well do you think this particular system looks at assessing those symptoms and issues that are of most importance to you personally?” ^b^Order based on combined total of first and second responses to respective questions

Of the scoring systems currently in use, POEM, SCORAD, and EASI were most popular among patients, although no single method was preferred by all patients (Fig. 4; Table S2).

POEM was seen as the most useful scoring method, with 63% of patients finding it useful to a great extent. Patients found this scoring system most helpful for understanding disease severity and providing a more complete approach to the assessment of symptom severity than other scoring systems.

However, some patients perceived the questions used in POEM to be too basic, stating that they did not explore the complexities of AD or consider the broader impact of AD on patients’ daily lives. In addition, patients were critical that symptoms relevant to all patients (e.g. skin redness) were missing from this scoring system, while symptoms relevant to only some patients with AD (e.g. skin bleeding) were included.

SCORAD was ranked as the second most helpful scoring system; 60% of patients found it useful to a great extent. As with POEM, patients liked that SCORAD covers the most reported symptoms. In addition, patients praised the scoring system’s collaborative approach to assessing patients’ needs, stating that they were more likely to find the most suitable treatment if they felt they were working with their clinician toward the best outcome. However, patient feedback highlighted SCORAD’s limitations relating to the impact of AD on daily activities, pain, bleeding, and weeping skin. For some patients, their AD did not affect an accessible place on their body, and these patients found SCORAD to be of limited benefit.

EASI was reported to be the third most useful scoring system, with 56% of patients finding it helpful to a great extent. This scoring system received positive feedback regarding its comprehensive questions; patients felt that it provided a good indication of the physical impact of living with AD.

However, this system was perceived as less relevant to patients suffering from mild AD, for whom the impact of psychological symptoms was greater than the impact of physical symptoms. Another issue highlighted by patients was that EASI can sometimes provide a skewed result if scores are high in one area and low in others. Patients also did not perceive EASI to account for differences in AD severity across different areas of the body or the subsequent impact on patients’ lives.

Patients indicated that an optimal scoring system should cover a range of symptoms and consider the variable nature of AD. In addition, patients reported that the ideal system must be accessible regardless of education level and help patients communicate the burden of AD to their clinicians, thus providing a clear framework for treatment.

Patients’ feedback indicated that they would like AD scoring systems incorporated into clinical practice to help them communicate their disease burden to clinicians and provide a clear framework for monitoring treatment response. Clinician objectivity was particularly important to patients, as they felt that some patients might exaggerate their symptoms to receive more effective treatment. Patients were also concerned that this may result in clinicians underestimating the symptoms of “truthful” patients.

## Discussion

This global patient research study generated insights into the burden of AD on patients’ lives, their expectations of treatment, and how patients make treatment decisions. In addition, it captured patients’ perspectives on AD scoring systems, what they thought was important to consider in an ideal system, and their experiences with their clinicians. The data showed that many patients felt that their clinicians underestimated the burden of AD and patients reported an inability to communicate optimally with them during consultations. Moreover, patients reported not having enough time to express themselves during medical appointments. Patient concerns regarding communication with their clinicians were also highlighted in their views on scoring systems, which they generally perceived as clinician-centric, prioritizing helping clinicians to evaluate their skin, rather than allowing patients to communicate their concerns.

A key strength of the study is the in-depth qualitative data generated–patients were encouraged to provide comments and additional detail when answering questions. This allowed patients to elaborate on the multidimensional burden of their disease and to provide insights outside the scope of a limited set of questions. Additionally, the sample comprised a global, ethnically diverse range of patients. However, only adult patients (≥ 18 years old) were interviewed; therefore, this study does not generate insights relating to the unique burden and needs of adolescents or children suffering from AD. In addition, further countries could have been included to more comprehensively capture global patient perspectives. For example, although patients from Latin America participated in this study, only two Latin American countries were represented: Mexico (*N* = 5) and Brazil (*N* = 5). Only one country in the Middle East was represented (Saudi Arabia [*N* = 5]), and no patients from Africa or the South Asian continent were included.

Data from this study are consistent with previous findings [4–6] in highlighting the substantial disease burden of AD. Itch, skin redness, dry/flaky skin, and sleep disturbance were the symptoms most frequently reported by participating patients. These responses are in line with the findings of a recent literature review, which found that itch and psychological symptoms (e.g. sleep disturbance, anxiety, depression) were the most frequently reported symptoms of AD [3]. However, the use of free-form questions and prompting patients to provide additional detail in their answers generated new insights into patients’ views of their AD management.

Data from this qualitative study highlight the importance of patient–clinician communication, being integral to clinical practice and essential in building a good interpersonal relationship [13]. A review of qualitative research and questionnaires on what encapsulates a “good doctor” revealed that while clinicians emphasized strong medical skills, patients focused on good communication [14]. Those results show an unmet need for a treatment decision-making model that will help patients more effectively communicate their disease burden and enable clinicians to optimally treat their patients. Shared decision-making models, whereby patients are encouraged to ask questions and make informed treatment decisions, are increasingly used in modern clinical practice. It is, therefore, important that patients feel comfortable when communicating with their clinicians to achieve optimal outcomes. Optimal clinician–patient communication has been shown to result in better therapeutic outcomes for patients [15], and research suggests that it is not an innate skill but rather one that can be developed and enhanced with practice [16]. However, efforts to develop these skills predominantly occur in medical school, and the communication skills of busy clinicians often remain underdeveloped [15]. Therefore, continual efforts to improve clinicians’ communication skills should be encouraged.

This global study generated insights into the impact of AD symptoms on patients’ daily lives, how patients make treatment decisions, patients’ treatment expectations, their communication with clinicians, and their views on scoring systems. Patient feedback on their experiences with clinicians and the perceived barriers they encounter in medical appointments will help clinicians improve their practice. Finally, the results provide evidence to support the creation of guidelines and recommendations that are patient-centric and aim to improve care standards [17].

## Electronic supplementary material

Below is the link to the electronic supplementary material.

Supplementary Material 1

## Data Availability

The data underlying this article are available in the article and in its online supplementary material.
