# Supplementary material for: Patient-reported burden in adults with atopic dermatitis: an international qualitative study
Source: Arch Dermatol Res. 2024 Jun 8;316(7):380. doi: 10.1007/s00403-024-03130-w (PMC11162389; doi:10.1007/s00403-024-03130-w)
Supplement: Supplementary file 1 — Supplementary Material 1 [file 403_2024_3130_MOESM1_ESM.docx]

**Patient-reported burden in adults with atopic dermatitis: an international qualitative study**

Archives of Dermatological Research

Andreas Wollenberg^1,2^ · Melinda Gooderham^3,4^ · Norito Katoh^5^ · Valeria Aoki^6^ · Andrew E. Pink^7^ · Yousef Binamer^8,9^ · Jonathan I. Silverberg^10^

^1^Department Dermatology and Allergy, Ludwig-Maximilian University of Munich, Munich, Germany

^2^Department of Dermatology, Augsburg University Hospital, Augsburg, Germany

^3^Department of Dermatology, Queen’s University, Ontario, Canada

^4^Department of Dermatology, SKiN Centre for Dermatology, Ontario, Canada

^5^Department of Dermatology, Kyoto Prefectural University of Medicine, Kyoto, Japan

^6^Department of Dermatology, University of São Paulo School of Medicine, São Paulo, Brazil

^7^St. John’s Institute of Dermatology, Guy’s & St. Thomas’ NHS Foundation Trust, London, UK

^8^Department of Dermatology, Alfaisal University, Riyadh, Saudi Arabia

^9^Department of Dermatology, King Faisal Specialist Hospital & Research Centre, Riyadh, Saudi Arabia

^10^George Washington University School of Medicine and Health Sciences, Washington DC, USA

**Correspondence to:**

Andreas Wollenberg

[wollenberg@lrz.uni-muenchen.de](mailto:wollenberg@lrz.uni-muenchen.de)

**Supporting information**

| Table S1 Patient-friendly descriptions of AD severity scoring systems | |
| --- | --- |
| Outcome measure | Patient-friendly description |
| Patient-reported measures |  |
| Itch NRS | Patients rate the itchiness of their eczema on a scale from 0 (no itch) to 10 (worst imaginable itch). One might record either an average itch or worst-possible itch with this scale |
| Sleep NRS | Patients rate the sleeplessness caused by their eczema on a scale from 0 (no sleeplessness) to 10 (worst imaginable sleeplessness) |
| Pain NRS | Patients rate the pain caused by their eczema on a scale from 0 (no pain) to 10 (worst imaginable pain) |
| DLQI | Patients complete a questionnaire about the effects of their skin disease on their quality of life. It includes 10 questions covering subjects such as sport and leisure activities, working and studying, sexual difficulties, and self-consciousness |
| POEM | Patients answer seven questions, each about a different symptom of eczema. The symptoms covered are itchiness, sleeplessness, bleeding skin, weeping or oozing skin, cracked skin, flaky skin, and dry or rough skin |
| WPAI | Patients answer six questions about the effect of eczema on their work and activities. The questions cover work time missed, loss of productivity when at work, and effects on activities other than work (such as shopping, childcare, and exercise) |
| MTBQ | Patients complete a questionnaire about how many medications they are taking and the effect this has on their quality of life |
| Patient-reported global AD severity | Patients provide an overall rating of the severity of their eczema |
| Clinician-reported measures | |
| BSA | Doctors examine the skin and measure the percentage area of skin affected by eczema. The severity of eczema lesions is not measured |
| IGA | Doctors rate the eczema on a 5-point scale: 0 (clear), 1 (almost clear), 2 (mild), 3 (moderate), or 4 (severe). This is based on factors such as redness, oozing, and crusting in the skin |
| EASI | Doctors measure both the area of skin affected by and the severity of eczema. Severity includes redness, thickness, and scratching |
| SCORAD | Like for EASI, doctors measure the area of skin affected by and the severity of eczema. In addition, SCORAD includes itch, dry skin, and sleeplessness, all scored by the patient |
| Rajka–Langeland score | Doctors measure the area of skin affected by eczema, and patients report the itchiness of their eczema and the course of their disease (ie, whether eczema is always present or if they have periods when it disappears) |

*AD* atopic dermatitis, *BSA* body surface area, *DLQI* Dermatology Life Quality Index, *EASI* Eczema Area and Severity Index, *IGA* Investigator’s Global Assessment, *MTBQ* Multimorbidity Treatment Burden Questionnaire, *NRS* Numeric Rating Scale, *POEM* Patient-Oriented Eczema Measure, *SCORAD* SCORing Atopic Dermatitis, *WPAI* Work Productivity and Activity Impairment

| Table S2 Key quotes from interviews |
| --- |
| Impact of AD on patients’ lives and patients’ most troublesome symptoms |
| ‘I didn’t feel that I wanted to just go to work; I didn’t want to engage in any social events with friends and I just wanted to stay confined in my house.’ |
| ‘I had such a severe itch a year ago that I scratched so much that it led to blood poisoning and a week and a half in hospital.’ |
| ‘If you do not sleep, it immediately leads onto psychological impacts – the physical and the psychological are linked.’ |
| ‘Sleep disturbance, itching, weeping and mental health are all interrelated and the impact from one affects the others.’ |
| How patients make treatment decisions |
| ‘Itching that was maddening, that you wanted to rip your skin off, and the itching caused scratching, wounds that bled and were especially painful, and the skin was still itching despite scratching off the upper layer of the epidermis.’ |
| Patients’ treatment expectations and communication with clinicians |
| ‘I’ve come to set my expectations kind of low with eczema.’ |
| ‘The treatment has reduced the frequency in which I experience itch – I no longer have it every day, several times per day. However, it has not eliminated it completely, which would be ideal, and the intensity of the flare-ups is the same as before.’ |
| ‘I feel like they wouldn’t really listen to what I was trying to say; they would take one look at my skin, make their own assumptions and treat me based on that, and it was more of a lecture that I would sit and have to listen to, rather than a conversation where I actually contribute.’ |
| Patients’ views on AD scoring systems |
| ‘My doctor has never used any scales or scoring systems with me; I have never filled one out.’ |
| ‘I don’t agree with scales; it is more for doctors.’ |
| ‘I think both [patient- and clinician-reported outcomes] are quite important to have because a doctor looking at it might see something different that you’ve just grown used to, but I think it’s also quite important to get the patient’s point of view on how it affects them and their life and what’s going on for them.’ |
| ‘I think the best thing would be to use both of them [patient- and clinician-reported outcomes], one of each, so that way you get an idea of the actual effect on patients, but that would be coupled with the professional opinion and a slight objective opinion on the condition. So, it needs a mixture of both.’ |
| ‘It [POEM] breaks it down by symptom, and you have a chance to respond differently depending on the symptoms.’ |
| ‘It [SCORAD] covers the three main big problems that I experience: itch, dry skin and sleeplessness. This is what eczema is to me.’ |
| ‘This [EASI] is good for keeping track, but it’s really just about how the skin looks, which they can see. It doesn’t add anything.’ |
| ‘Any system would be of no value unless the patient is advised to keep track of it for a month like you measure blood pressure. But if that’s only on the day of a doctor’s appointment, say once a month, what if they happen to be having a good day or having a bad day?’ |

*AD* atopic dermatitis, *EASI* Eczema Area and Severity Index, *POEM* Patient-Oriented Eczema Measure, *SCORAD* SCORing Atopic Dermatitis
